# Enhancing Workforce Capacity to Improve Vaccination Data Quality, Uganda

**DOI:** 10.3201/eid2313.170627

**Published:** 2017-12

**Authors:** Kirsten Ward, Kevin Mugenyi, Amalia Benke, Henry Luzze, Carol Kyozira, Ampeire Immaculate, Patricia Tanifum, Annet Kisakye, Peter Bloland, Adam MacNeil

**Affiliations:** Centers for Disease Control and Prevention, Atlanta, Georgia, USA (K. Ward, A. Benke, P. Tanifum, P. Bloland, A. MacNeil);; African Field Epidemiology Network Secretariat, Kampala, Uganda (K. Mugenyi);; Ministry of Health, Kampala (H. Luzze, C. Kyozira, A. Immaculate);; World Health Organization, Kampala (A. Kisakye)

**Keywords:** vaccination, immunization, data quality, information systems, workforce development, program evaluation, Uganda, global health security, vaccines

## Abstract

In Uganda, vaccine dose administration data are often not available or are of insufficient quality to optimally plan, monitor, and evaluate program performance. A collaboration of partners aimed to address these key issues by deploying data improvement teams (DITs) to improve data collection, management, analysis, and use in district health offices and health facilities. During November 2014–September 2016, DITs visited all districts and 89% of health facilities in Uganda. DITs identified gaps in awareness and processes, assessed accuracy of data, and provided on-the-job training to strengthen systems and improve healthcare workers’ knowledge and skills in data quality. Inaccurate data were observed primarily at the health facility level. Improvements in data management and collection practices were observed, although routine follow-up and accountability will be needed to sustain change. The DIT strategy offers a useful approach to enhancing the quality of health data.

Optimal immunization coverage against vaccine-preventable diseases (VPDs) is essential for achieving and maintaining global health security. Obtaining such coverage relies on high-quality immunization data, which are a prerequisite for good decision making; effective and efficient public health action, monitoring, and evaluation; and improved population immunity against VPDs (*1**–**3*). Enhanced demand for vaccination data and scrutiny of their quality are evident in strategic guidance documents for the Global Polio Eradication Initiative (GPEI) (*4*), the Global Vaccine Action Plan (*5*), and the recently introduced data quality requirements for financial support from Gavi, the Vaccine Alliance (*6*). Availability and quality of vaccination data are often inadequate to inform policy, effective management, and monitoring of vaccination programs (*3**,**7**,**8*).

In 2013, Uganda conducted a national data quality self-assessment (DQS) (*9*) (Ministry of Health, Uganda, unpub. data) and found that the quality of administrative vaccination data was suboptimal, particularly at the subnational level, which was likely contributing to inflation of administrative coverage data (*10*). Reasons for poor data quality included inaccurate vaccine dose administration data generated at the health facility, deficiencies in healthcare worker knowledge and skills, scarcity of standard recording and reporting tools, and inadequate implementation of recommended practices for data management collection, analysis, and use. Many of these issues had been previously identified in Uganda and elsewhere (*8**,**10**–**12*). To guide implementation of recommendations from the DQS, the technical working group for the Ugandan National Expanded Program on Immunization (UNEPI) developed the National Data Quality Improvement Plan. This plan laid out how, and at what level, the recommendations would be addressed, recognizing limited published evidence regarding effectiveness of specific approaches to strengthen immunization data quality (*3**,**12**,**13*). Given the importance of an effective workforce, a central component of the Data Quality Improvement Plan was to enhance the capacity of existing healthcare workers to manage, analyze, and use vaccination data. The chosen approach was guided by growing evidence supporting on-the-job training of healthcare workers that includes feedback and follow-up (*14*), which had previously been used successfully in Uganda (*15**,**16*). This article describes the initial implementation (November 2014–September 2016) and outcomes of Uganda’s national strategy to improve administrative vaccination data quality, defined by the dimensions of management; collection; data produced (accuracy, timeliness, completeness); analysis; and use (*17*).

## Methods

### Preparation for the Data Improvement Team Strategy

The data improvement team (DIT) strategy was developed and managed by a national DIT strategy management group, which included UNEPI, the Resource Center (the responsible entity for managing health information) of the Uganda Ministry of Health, World Health Organization (WHO) Uganda, the US Centers for Disease Control and Prevention (CDC), the African Field Epidemiology Network (AFENET), UNICEF, and Gavi. Implementation was funded jointly by Gavi Health Systems Strengthening Grant 1, WHO, UNICEF, and CDC and led by a national coordinator from AFENET, with technical assistance from CDC.

**Figure 1:**
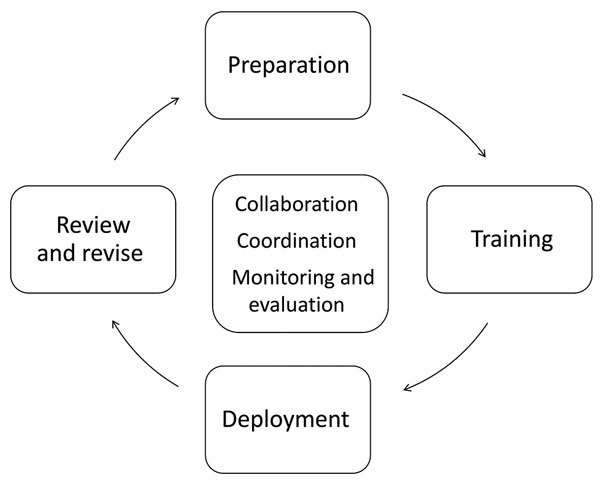
Overview of processes and key activities of the Uganda data improvement team (DIT) strategy to improve vaccination data quality. At the center are elements that are ongoing throughout implementation of the 4 main activities: financial, technical, and logistical collaboration between Expanded Program on Immunization partners, coordination provided by a DIT strategy management group and the DIT national coordinator, and routine monitoring and evaluation. Preparation includes discussing and developing budget, designing the approach to implementation and materials for training and monitoring and evaluation, training supervisors, grouping districts into regions, and identifying DIT members. For training, grouped by region, DIT members from several districts attend a 3-day training led by staff from the Ministry of Health Expanded Program on Immunization and the DIT strategy national coordinator. This training included a combination of technical lectures, practical case studies (80% of all sessions), and a practice visit to a health facility (half-day). Deployment core activities include district and health facility organizational assessment and a rapid data quality improvement questionnaire to identify strengths and gaps in resources and systems for immunization data management, collection, analysis, and use. Results inform recommendations developed by the DIT members who provide on-the-job training of staff to strengthen action on recommendations. DIT members debrief leadership (region, district, health facility) on findings and recommendations, and harness support to implement recommendations. Finally, national DIT strategy management groups review activities and results at several time points (Figure 2); based on evidence from implementation and current national priorities, the strategic and operational approaches are revised, then reimplemented.

The strategy aimed to strengthen the immunization information system and quality of the resultant data at the district and health facility levels through practical classroom training, deployments involving rapid data quality and organizational assessments, and on-the-job training (Figure 1). The number of DIT members required for each district was determined on the basis of ability to reach all health facilities that provided immunization services in that district (range 6–117) within 5 to 6 working days, spending 2 to 3 hours at each. A district-level DIT included an average of 4 district staff members (with additional members in high-population areas) and 1 Makerere University School of Public Health (MakSPH) student. Districts were asked to identify staff to form a DIT, which included the district biostatistician, district Expanded Programme on Immunization (EPI) and surveillance focal persons, and a health records assistant. MakSPH staff and the national DIT coordinator led recruitment of students.

**Figure 2:**
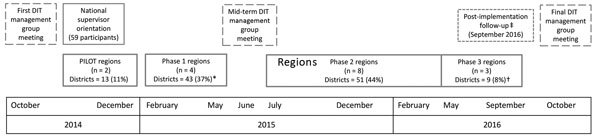
Implementation timeline for the DIT strategy to improve vaccination data quality, Uganda, 2014–2016. Systematic comparison of the number of doses of vaccine recorded on the paper-based monthly HMIS report and the electronic HMIS data was conducted only in the first 48% (n = 56) of districts where the DIT strategy was implemented. *Design of training curriculum changed to enhance delivery through case-study–based and practical sessions. Additional content was added in the following areas: monitoring and evaluation activities for the DIT strategy, supportive supervision, and development of specific, measurable, achievable, realistic and time-bound recommendations. †Mobile application introduced for DIT members to report results from organizational assessment and data quality improvement questionnaire. ‡Postimplementation review conducted in sample of districts and health facilities. DIT, data improvement team; HMIS, Health Management Information System

The DIT strategy was designed to be implemented in a phased approach by region (Figure 2); several district-level DITs were trained together, then deployed in their respective districts. All official government districts in Uganda as of November 2014 were divided into 17 DIT operational regions to ensure that the number of attendees at regional training was logistically manageable and there was close geographic proximity between districts in each region.

### Training

Before implementation, a 5-day orientation to the strategy and Uganda’s immunization information systems was provided to national staff, who self-selected to support delivery of the regional-level training and to conduct supportive supervision of DIT activities. The 3-day regional training aimed to build selected DIT members’ knowledge and skills in data management and quality, which were applicable during the DIT deployment and their regular duties thereafter (Figure 1).

### Deployment

In the week after each regional training, DIT members were deployed to their home districts for 5 to 6 days to work at the district office and visit health facilities (Figure 1). Working in pairs, DIT members identified problems, proposed solutions, developed recommendations, and enhanced staff capacity through on-the-job training on locally identified problems (e.g., how to create an immunization monitoring chart) (Figure 2). DITs initially prioritized health facilities with outlying (high or low) coverage for the third dose of the diphtheria/tetanus/pertussis/*Haemophilus influenzae* type B/hepatitis B vaccine (Penta3), negative dropout rates, or inadequate completeness or timeliness of Health Management Information System (HMIS) monthly reports (*18*). Staff from the Ministry of Health and national EPI program partner organizations provided supportive supervision to DIT activities in some districts, assisting coordination and implementation of activities, conveying national-level support for the DIT strategy to district leadership, and enhancing their own awareness of ground-level operations.

### Monitoring and Evaluation

A participatory and utilization-focused (*19**,**20*) approach was taken to routine monitoring and evaluation of processes, outputs, and short-term outcomes. Training was evaluated through a self-administered survey focused on quality of the training experience; a pretest and posttest measured participants’ acquisition of knowledge and level of preparedness to implement DIT activities. DITs conducted an organizational assessment at the district and health facility levels to inform their work and to gather baseline information on key indicators (*21*). Organizational assessments contained a mix of closed and open questions covering dimensions of, and factors affecting, vaccination data quality. Results of organizational assessments were reported to the DIT national coordinator either through a reporting template in Excel (Microsoft, Redmond, WA, USA) (106 districts, 1 Kampala division) or by using an open data kit–based mobile application linked to a cloud-based database (5 districts, 4 Kampala divisions).

At the health facility, DITs also used a data quality improvement (DQI) questionnaire to review practices for data management, collection, accuracy, analysis, and use (Table 1). The primary purpose of this questionnaire was to identify gaps that would inform recommendations and on-the-job training. For purposes of analysis for monitoring and evaluation, DQI questionnaires from health facilities were sampled from 107 of the 116 districts (92%) for which these data were not reported through the mobile application. The sample included all hospitals and every second health facility selected from an alphabetized list, until the sample size reached 50% of all health facilities in the district. In an additional 7 districts, DQI reports from all visited health facilities were entered in the mobile application. Descriptions of the DIT activities, outputs, and recommendations were presented in a written report for each district health management team. Line-listed results from organizational assessments and DQI questionnaires were aggregated nationally and quantitative data were descriptively analyzed in SAS version 9.3 (*22*) and Tableau version 9.3.1 (*23*). The sign test was used to assess the statistical significance of the direction of difference between sources of vaccine dose administration data and was performed in R version 1.5.1 (*24*).

**Table 1:** Reach and key observations in district and health facilities from the first phase of the data improvement team strategy to improve vaccination data quality in Uganda*

Data quality domain	Description	Districts, no. (%)	Health facilities, no. (%)
DIT strategy reach	District and health subdistrict staff trained	454 (NC)	NC
	District and health subdistrict staff deployed as DIT members	441 (NC)	NC
	Districts reached	116 (100)*	NA
	Districts where harmonization of monthly report and DHIS2 data conducted	48 (56)*	NA
	Health facilities (that provided immunization services) reached	NC	3,443 (89)†
Knowledge and practices			
Collection	Process for incorporating late HMIS monthly reports (HMIS105) into the DHIS2	98 (84)‡	NC
	Known (documented) target population <1 y of age	NC	1,797 (53)§
	Demonstrated use of immunization data recording and reporting tool		
	Child register	NC	2,713 (78)§
	Tally sheet	NC	2,847 (84)§
	HMIS monthly report forms	NC	3,086 (91)§
	Vaccine control books	NC	1,980 (58)§
Analysis	Monthly immunization coverage for Penta3 charted on a monitoring chart	NC	1,099 (32)§
	Monitoring chart of immunization coverage for Penta3 displayed	NC	1,153 (34)§
Use	Demonstrated use of immunization data to inform action	79 (68)‡	1,503 (44)¶
Management	Old copies of immunization data are archived in an organized and easy-to-locate manner		
	Child register	NC	2,367 (70)§
	Tally sheet	NC	2,239 (66)§
	HMIS monthly report forms	87 (75)‡	2,455 (72)§
	External factors		
Collection + analysis + use	Inability to access the DHIS2 in >1 month in the 3 months before DIT visit	56 (48)‡	NC
Management + collection + analysis + use	Presence of specific roles# responsible for immunization data management and reporting	107 (92)‡	1,399 (41)¶
Collection	Blank copies of immunization data collection tools available at time of DIT visit		
	Child register	NC	1704 (50)§
	Tally sheet	NC	2,459 (72)§
	HMIS monthly report forms	NC	1,706 (50)§
	Vaccine control books	NC	1,806 (53)§

*A total of 112 districts plus the 5 Kampala divisions each were considered a separate district for DIT strategy operational purposes. Total DIT strategy operational districts = 116. Data from Ugandan Ministry of Health, November 2014. DHIS, District Health Information System; DIT, data improvement team; HMIS, Health Management Information System; NA, not applicable; NC, not calculated; Penta3, diphtheria/tetanus/pertussis/*Haemophilus influenzae* type b/hepatitis B vaccine, third dose. †Of 3,856 health facilities that provide immunization services, identified by the DITs at time of visit. ‡Of 116 DIT strategy districts where the DIT district checklist was completed during deployment. §Of 3,392 health facilities where the data quality improvement tool was completed by DITs. ¶Of 3,443 health facilities where the health facility checklist was completed by DITs. #At district, these roles included an HMIS focal person or biostatistician. At health facility, roles included health records assistant or health information assistant.

After DITs had been deployed to all districts, a review of DIT implementation was undertaken to gather feedback about the approach and understand extent of action on recommendations through a rapid organizational-level survey in a sample of districts and health facilities. Four regions were selected from the 17 DIT operational regions; 2 or 3 districts were selected from each region, and within each of these, 4 health facilities were selected, totaling 11 districts and 44 health facilities. If a selected site could not be visited, it was replaced with the next one of the same type on an alphabetized list of health facilities in the district. Selection was purposeful to gain insights across a range of characteristics, including geographic location, implementation of national supervision, Reaching Every District categories (*25*), and level (type) of health facility (*26*). Eight data collectors (4 AFENET/CDC staff and 4 MakSPH students) completed a 1-day training, then worked in pairs to visit the selected sites to conduct the survey through group interviews with district and health facility staff. Resultant data were descriptively analyzed in Epi Info software (*27*).

The proportion of health facilities in a district submitting monthly HMIS reports on time to the district (timeliness) and the proportion of expected reports received by the district (completeness) are routinely calculated in the national electronic HMIS (*12**,**18*). In districts for which these data were available for the 3 months and after the DIT visit (n = 104) and for the second 3-month period after the DIT visit (n = 95), timeliness and completeness, by month and district, were extracted from the electronic HMIS. Median timeliness and completeness were calculated per district across each 3-month period, then compared between periods to identify change.

### Review and Revision

The national DIT strategy management group held periodic meetings (Figure 2) to review results from monitoring and evaluation and the budget, as well as to solicit feedback from all stakeholders. These meetings, in conjunction with national priorities, informed any adjustment of DIT activities and implementation plan.

## Results

### Training and Deployment

During November 2014–September 2016, all 112 districts and 5 divisions of Kampala (total 116 DIT operational districts) in Uganda sent staff to DIT regional training and deployed district-level DITs. Seventeen regional trainings, covering 2–14 districts per training, attended by 451 district and health subdistrict staff and 35 MakSPH students (some attended multiple trainings [range 1–9]). In response to participant and stakeholder feedback, the training format was altered to enhance the balance between the practical and didactic sessions (Figure 2). After training, 83% (355/429) of district staff demonstrated improved knowledge on posttest compared with pretest scores, and more participants felt “fully prepared” to conduct DIT activities (14% pretest, 82% posttest).

**Figure 3:**
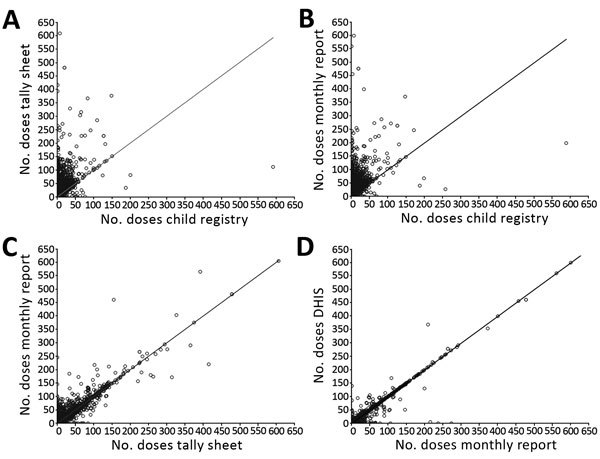
Comparison of the number of doses of Penta3 recorded on different vaccine dose recording and reporting tools, Uganda. A) Doses recorded on tally sheet compared with immunization register (n = 1,664 health facilities); B) doses recorded on monthly report compared with immunization register (n = 1,686 health facilities); C) doses recorded on monthly report compared with tally sheet (n = 1,713 health facilities); D) doses recorded on the compared with monthly report (n = 1,661 health facilities; 3 outliers not shown [total no. doses >650]). p<0.001 for all comparisons. Data from sample of 2015 DQI tools; 1,667 (83%) sampled from 107 districts and 343 (17%) from a census of 7 districts. Data were missing from 2 districts. DHIS, District Health Information System; Penta3, diphtheria/tetanus/pertussis/*Haemophilus influenzae* type b/hepatitis B vaccine, third dose.

In total, 476 DIT members (including 35 MakSPH students) were deployed and reached 89% of health facilities that provided immunization services (Table 1). Health facilities not visited (n = 413) were predominantly health center IIs (HCIIs; n = 332, 80%), which offer a limited number of services, serve smaller catchment areas, and are often geographically remote. Initially, DITs reviewed paper copies of monthly HMIS reports from health facilities submitted to the district office and compared doses reported for all antigens with those recorded in the electronic HMIS for the 12 months before the DIT visit (Table 1). Time spent on this activity reduced the time available to reach all priority health facilities by an average of 8 hours per district. Because early results showed high congruence between these 2 data sources (Figure 3, panel D), this activity ceased after the midterm review meeting, enabling DITs additional time to conduct organizational and DQI assessments and develop recommendations for improvement (average 1.2 hours per health facility) and to implement on-the-job training (average 1.5 hours per health facility).

Through the organizational assessment, DQI questionnaire, and discussions with staff, DITs identified a combination of external factors, often specific to the site visited, that affected vaccination data collection, management, analysis, and use. Commonly identified challenges included poorly motivated, new, untrained, or absent staff; unavailability of materials for recording and reporting data; competing priorities on staff time due to integration of services; inadequate supportive supervision for data quality; limited transport, technological, and financial resources; variable understanding and commitment by political or organizational leaders; and competition with other public health initiatives for human, financial, and material resources.

In Uganda, doses of vaccines administered are recorded on 4 tools: tally sheet, child register, and monthly report (at health facility) and the electronic HMIS (entered at district level using data from health facilities’ monthly report). We found variable congruence between monthly totals of vaccine doses across these 4 sources for any given month (Figure 3). On average, the number of administered doses aggregated on the monthly report was higher than that recorded individually on the tally sheet (Figure 3, panel C), which was higher than that recorded on the child register (Figure 3, panel A). This finding suggests that vaccine administration is overreported by the health facility and that use of the child register is low compared with other sources of vaccine dose administration data (Figure 3, panels A, B). We found stronger agreement between the number of doses on the paper HMIS monthly report and those in the electronic HMIS (Figure 3, panel D), highlighting infrequent transcription error or loss of data from district to national level. There was individual variation in the discordance by health facility, with no clear pattern by district or health facility type. Similar patterns in data congruence were also seen for single-dose measles vaccine offered to older children (data not shown).

### Postimplementation Follow-Up

The postimplementation follow-up survey found that DITs had visited all sampled districts (n = 11) and 77% (34/44) of sampled health facilities. Recommendations provided by DITs addressed all dimensions of data quality; however, the extent of implementation varied (Table 2). Recommendations for each district most frequently related to improving systems for archiving, checking data on monthly HMIS reports, and charting coverage data. At the district level, recommendations relating to data management and collection were more fully implemented than those related to analysis and use (Table 2). Recommendations for health facilities most commonly focused on improving recording and reporting of data, analysis, and archiving. Recommendations related to management and collection were more completely implemented than those related to analysis. No health facility reported taking action on recommendations to improve data use (Table 2). Reasons for inaction across all recommendations included insufficient availability of required materials (standard data collection/reporting tools, archiving space); inadequate human resource capacity (new staff, untrained staff, low motivation); and a management structure that limited staff awareness of, and roles in, immunization data collection, management, analysis, and use.

**Table 2:** Key themes from DIT recommendations to improve vaccination data quality and extent of implementation of these at follow-up in select districts (n = 11) and health facilities (n = 34) in Uganda*

Theme of recommendations	Districts, no. (%), n = 11					Health facilities, no. (%), n = 34			
Completely implemented	Partially implemented	Not at all implemented	Unable to determine	Completely implemented	Partially implemented	Not at all implemented	Unable to determine
Analysis and use of EPI data, including monitoring charts	2 (22)	1 (11)	6 (67)	0		8 (32)	9 (66)	8 (32)	0
Archiving of data	3 (38)	3 (38)	1 (12)	1 (12)		11 (61)	5 (28)	2 (11)	0
Meetings to review results	0	0	1 (50)	1 (50)		†	†	†	†
Recording and reporting of data	1 (20)	1 (20)	2 (40)	1 (20)		16 (49)	6 (18)	7 (21)	4 (12)
Systems for review/checking of reported data	3 (43)	0	3 (43)	1 (14)		2 (40)	2 (40)	1 (20)	0
Use of immunization data for decision making	1 (50)	1 (50)	0	0		0	0	5 (100)	0
Improve accuracy and knowledge of catchment area population	†	†	†	†		1 (50)	0	1 (50)	0

*District and health facilities visited during post-implementation follow-up which showed evidence of visit from DIT. DIT, data improvement team; EPI, Expanded Programme on Immunization. †Theme not identified at this level.

During the follow-up survey, district staff frequently reported that participation in the DIT activities catalyzed improvements in existing, or development of new, systems and processes, such as supportive supervision about vaccination data quality. Health facility staff felt that the visit by the DITs was a catalyst for provision of updated recording and reporting tools and helped them develop systems to enhance completeness and accuracy of data reported on the monthly HMIS report (Table 3).

**Table 3:** Extent of self-reported changes catalyzed by the DIT visit to improve vaccination data quality in select districts and health facilities in Uganda*

Area of change	No. (%) districts reporting change, n = 11	No. (%) health facilities reporting change, n = 34
Supportive supervision visits include review and follow-up on quality of vaccination data	9 (82)	†
Routine checking of accuracy of data entered into the DHIS2	8 (73)	†
Checking completeness and accuracy of monthly report data before acceptance	8 (73)	†
Analysis and use of data	6 (55)	†
Archiving of data	6 (55)	†
Changes in supply of recording and reporting tools	†	18 (53)
Checking monthly report data with primary data source	†	18 (53)
Improved practice in recording data on tally sheets and child register	†	50 (17)
Analysis and use of immunization data	†	47 (16)

*District and health facilities visited during postimplementation follow-up that showed evidence of visit from DIT. DHIS, District Health Information System; DIT, data improvement team. †Change in this area not reported at this level.

Timeliness and completeness of HMIS monthly reporting (from health facility to district) averages >90% nationally (*28*). This high performance limits the opportunity for and measurement of change; however, there was some improvement. Comparing 3 months before and after DIT implementation, 15% (15/104) of districts showed improvement in completeness, 6% (10/104) decreased completeness, and the remainder no change. From the initial 3 months to the second 3 months post-DIT implementation, completeness improved in 25% (24/95) of districts, decreased in 10% (9/95), and showed no change in the remainder. More districts showed improvement in timeliness of monthly HMIS reporting. Comparing 3 months before DIT implementation to 3 months after, 38% (40/104) improved, 20% (21/104) decreased, and the remainder showed no change in timeliness. From the first to second 3-month periods after implementation, 27% (26/95) of districts showed improvement, 50% (47/95) decreased, and the remainder showed no change.

## Discussion

EPI partners in Uganda took a collaborative approach to developing, funding, and implementing a strategy to address recommendations from Uganda’s most recent DQS. Over 23 months, 351 district staff and 35 MakPSH students were trained and 479 DIT members were deployed, in phases, to all districts and 89% of health facilities that provide immunization services in Uganda. Rapid assessments of organizational-level immunization information systems and accuracy of resultant data identified gaps in skills and systems for data management, collection, analysis, and use. Assessments indicated that the child register was underused, and the tally sheet was used as the primary data recording tool, with greater variation in the difference between these primary data sources than for data aggregated at the district and national levels. Timeliness and completeness of HMIS monthly reports from health facilities was high at baseline; although some districts showed improvement, there was volatility in these changes. Recommendations for improvement and changes made by district and health facilities related predominantly to strengthening systems and processes, with those related to management and collection more completely implemented than those related to analysis and use.

DITs identified that poor data quality stemmed largely from inaccurate and incomplete recording and reporting of vaccine dose administration data at the health facility and poorly implemented processes for data management, collection, analysis, and use. These problems likely contributed to overreporting of administrative data, as identified in the 2013 Uganda DQS (*10*). If data are improperly recorded at, or inaccurately reported from, the health facility to the district level, these data will remain inaccurate in the national HMIS (*18*). Although data are prone to errors such as incorrect entry, incompleteness, or late reporting, accurate recording and reporting of vaccine doses administered from the initial point at which they are generated is critical to improving the quality and utility of data at all levels of the health system (*29*). The relationship between data quality and use could be considered cyclical, in that improving accuracy could improve confidence in the data, which would help drive demand and use, further driving data quality. At a service delivery level, if data are not used to monitor performance, opportunities can be missed to identify issues as they arise, such as problems with dropouts, changes in target population, or underserved areas, all of which can lead to underimmunized children and can leave the population vulnerable to outbreaks of epidemic-prone VPDs, which threatens global health security (*30*).

Improving data accuracy in a situation of overreporting may result in lower immunization coverage estimates (*7*). Despite implying poorer program performance, increased accuracy would enhance the utility of the data for informing immunization program implementation, including identification of underimmunized or nonimmunized populations that may have been masked by overreporting. Underrecording of individual-level vaccination status in the child register inhibits the ability of healthcare workers to identify and follow up with inadequately immunized children, both routinely to maximize coverage and during VPD outbreaks. Underrecording also reduces the utility of the child register as a secondary data source to verify caretakers’ recall when home-based vaccination records are not available (*31*). Home-based records enable health facility staff to routinely verify individual vaccination status and are critical to the success of periodic independent coverage surveys, which are valuable to verify administrative vaccination data. However, discordance between sources of data on vaccination coverage and inherent limitations in many sources of vaccination data make it difficult to determine true immunization coverage.

Some components of the DIT strategy are not typical of other approaches to national data quality improvement initiatives and could be applicable to other countries and other health data. First, the strategy was facilitated through a hybrid funding commitment across multiple organizations, which allowed it to be implemented nationally. Second, the combination of site-specific problem identification followed by immediate, on-the-job training was found to be a useful approach to strengthening healthcare workers’ awareness, knowledge, and skills. A similar package of interventions has been seen to improve the quality of supportive supervision for immunization in Georgia (*13*). Systematic literature reviews highlight the effectiveness of multifaceted approaches, which include audit, feedback, and supportive supervision, in building health workforce capacity (*14**,**32*). The capacity to understand the gaps and challenges faced and to tailor improvement strategies accordingly appears fundamental to improving immunization coverage (*33*). Third, involvement of existing national and district staff helped build sustainability. Finally, MakSPH students, many of whom were redeployed several times, developed their own knowledge and skills, which they felt enhanced their future job prospects. They also brought an external eye that enhanced problem detection, accountability, and external motivation of DITs and health facility staff.

There are limitations to individual methods used for monitoring and evaluation of the DIT strategy, although in combination they facilitated a better understanding about implementation and short-term change (*34*). DIT members and data collectors were trained in the use of data collection instruments, standard question prompts were included, and data validation was built into the mobile application. Systematic sampling of DQI tools for analysis reduced some systematic error and improved internal validity of these data. The magnitude of difference between sources of vaccination data was influenced by variation in month of assessment and number of doses reported, which was, in turn, a function of health facility type. Administrative data on timeliness and completeness of reported vaccination data are likely limited in specificity and internal validity. Feasibility influenced purposive selection of sites for the postimplementation follow-up, which was also open to researcher bias, although use of selection criteria helped reduce this (*35*). Different data collection methods were used for routine monitoring and postimplementation follow-up, which did not allow for extensive quantitative comparison between resultant data. Unless directly attributed through individual report, observed changes could not be credited solely to the DIT strategy.

Implementation of the first phase of the DIT strategy catalyzed stronger administrative vaccination data in Uganda. Informed by these experiences and results, a second round of DIT visits to all districts, targeting all health facilities, is being implemented. Planned modifications include follow-up to further determine extent of implementation of recommendations at all sites and degree of short-term change, as well as regular regional-level meetings of districts to improve accountability and drive action on recommendations. Assessment of vaccination data congruence will continue to focus on the health facility, although assessment of this across the immunization information system should be undertaken periodically to rule out any systematic data entry error or loss of data. The DIT strategy and observed changes have the potential to benefit data from other health initiatives, particularly those reported through the HMIS. Other countries looking to address vaccination data quality issues should consider a similar approach, using existing staff, on-the-job training, mechanisms for routine follow-up, and collaborative resource mobilization. Efforts should focus on identifying site-specific issues and building local workforce knowledge, skills, and awareness, as well as strengthening systems, to enhance availability, quality, and use of vaccination data.

## References

[1] World Health Organization, Health Metrics Network. Framework and standards for country health information systems. 2nd edition. Geneva: The Organization; 2008.

[2] O’Carroll PW, Yasnoff WA, Ward ME, Ripp LH, Martin EL. Public health informatics and information systems. New York: Springer Science+Business Media; 2003.

[3] Sodha SV, Dietz V. Strengthening routine immunization systems to improve global vaccination coverage. Br Med Bull. 2015;113:5–14. 10.1093/bmb/ldv001 25649959 PMC10350316

[4] Global Polio Eradication Initiative. Polio eradication and endgame strategic plan (2013–2018). Geneva: World Health Organization, 2013.

[5] World Health Organization. Global Vaccine Action Plan 2011–2020. Geneva: The Organization; 2012.

[6] Gavi, The Vaccine Alliance. General guidelines for applications for all types of Gavi support in 2016. 2016 [cited 2016 Dec 28]. http://www.gavi.org/library/gavi-documents/guidelines-and-forms/

[7] Strategic Advisory Group of Experts (SAGE). 2016 midterm review of the Global Vaccine Action Plan. Geneva: World Health Organization; 2016.

[8] Murray CJ, Shengelia B, Gupta N, Moussavi S, Tandon A, Thieren M. Validity of reported vaccination coverage in 45 countries. Lancet. 2003;362:1022–7. 10.1016/S0140-6736(03)14411-X 14522532

[9] Ronveaux O, Rickert D, Hadler S, Groom H, Lloyd J, Bchir A, et al. The immunization data quality audit: verifying the quality and consistency of immunization monitoring systems. Bull World Health Organ. 2005;83:503–10. 16175824 PMC2626295

[10] World Health Organization. Uganda: WHO and UNICEF estimates of immunization coverage: 2015 revision. 2016 [cited 2016 Dec 28]. http://www.who.int/immunization/monitoring_surveillance/data/uga.pdf

[11] Gavi The Vaccine Alliance. Joint Appraisal Uganda 2016. 2016 [cited Jan 04 2017]. http://www.gavi.org/country/uganda/documents/

[12] Kintu P, Nanyunja M, Nzabanita A, Magoola R. Development of HMIS in poor countries: Uganda as a case study. Health Pol Develop. 2005;3:46–53.

[13] Djibuti M, Gotsadze G, Zoidze A, Mataradze G, Esmail LC, Kohler JC. The role of supportive supervision on immunization program outcome - a randomized field trial from Georgia. BMC Int Health Hum Rights. 2009;9(Suppl 1):S11. 10.1186/1472-698X-9-S1-S11 19828055 PMC3226230

[14] Vasan A, Mabey DC, Chaudhri S, Brown Epstein HA, Lawn SD. Support and performance improvement for primary health care workers in low- and middle-income countries: a scoping review of intervention design and methods. Health Policy Plan. 2017;32:437–52. 27993961 10.1093/heapol/czw144 PMC5400115

[15] Cicciò L, Makumbi M, Sera D. An evaluation study on the relevance and effectiveness of training activities in Northern Uganda. Rural Remote Health. 2010;10:1250. 20170256

[16] Matovu JK, Wanyenze RK, Mawemuko S, Okui O, Bazeyo W, Serwadda D. Strengthening health workforce capacity through work-based training. BMC Int Health Hum Rights. 2013;13:8. 10.1186/1472-698X-13-8 23347473 PMC3565877

[17] Chen H, Hailey D, Wang N, Yu P. A review of data quality assessment methods for public health information systems. Int J Environ Res Public Health. 2014;11:5170–207. 10.3390/ijerph110505170 24830450 PMC4053886

[18] Kiberu VM, Matovu JK, Makumbi F, Kyozira C, Mukooyo E, Wanyenze RK. Strengthening district-based health reporting through the district health management information software system: the Ugandan experience. BMC Med Inform Decis Mak. 2014;14:40. 10.1186/1472-6947-14-40 24886567 PMC4030005

[19] Patton MQ. Essentials of utilization-focused evaluation. Los Angeles: SAGE; 2012.

[20] Guijt I. Methodological briefs: impact evaluation 5. Participatory approaches. Florence (Italy): UNICEF Office of Research; 2014.

[21] Simister N, Simth R. Praxis Paper 23: monitoring and evaluating capacity building: is it really that difficult? Oxford: International Training NGO Resource Center; 2010.

[22] SAS Institute. SAS Version 9.31M1. 2011 [cited 2017 Jan 4]. http://www.sas.com/en_us/home.html

[23] Tableau. Tableau Desktop Professional Edition. Version 9.3. 2016 [cited 2017 Jan 4]. http://www.tableau.com

[24] Yu Y, Yang T. signmedian.test: perform exact sign test and asymptotic sign test in large samples. R package version 1.5.1. 2015 [cited 2017 Mar 9]. https://CRAN.R-project.org/package=signmedian.test

[25] World Health Organization. The RED strategy. 2016 [cited 2017 Jan 6]. http://www.who.int/immunization/programmes_systems/service_delivery/red/en/

[26] Ministry of Health, Republic of Uganda. Uganda health facility master list—September 2016. Kampala (Uganda): Division of Health Information, Ministry of Health; 2016.

[27] Centers for Disease Control and Prevention. Epi Info Version 7. 2016 [cited 2016 Dec 28]. https://www.cdc.gov/epiinfo/index.html

[28] Ministry of Health Republic of Uganda HMIS Webportal. National reporting rates (all HMIS forms). 2017 [cited 2017 Feb 9]. http://hmis2.health.go.ug/#/

[29] Dunkle SE, Wallace AS, MacNeil A, Mustafa M, Gasasira A, Ali D, et al. Limitations of using administratively reported immunization data for monitoring routine immunization system performance in Nigeria. J Infect Dis. 2014;210(Suppl 1):S523–30. 10.1093/infdis/jiu373 25316876 PMC11037521

[30] Global Health Security Agenda. Immunization action package. 2017 [cited 2017 Mar 8]. https://www.GHSAgenda.org/packages/p4-immunization

[31] World Health Organization. World Health Organization vaccination coverage cluster surveys: reference manual. Version 3. Geneva: The Organization; 2015.

[32] Rowe AK, de Savigny D, Lanata CF, Victora CG. How can we achieve and maintain high-quality performance of health workers in low-resource settings? Lancet. 2005;366:1026–35. 10.1016/S0140-6736(05)67028-6 16168785

[33] LaFond A, Kanagat N, Steinglass R, Fields R, Sequeira J, Mookherji S. Drivers of routine immunization coverage improvement in Africa: findings from district-level case studies. Health Policy Plan. 2015;30:298–308. 10.1093/heapol/czu011 24615431 PMC4353894

[34] Bamberger M, Rugh J, Mabry L. Real world evaluation, 2nd edition. London: SAGE; 2014.

[35] Palinkas LA, Horwitz SM, Green CA, Wisdom JP, Duan N, Hoagwood K. Purposeful sampling for qualitative data collection and analysis in mixed method implementation research. Adm Policy Ment Health. 2015;42:533–44. 10.1007/s10488-013-0528-y 24193818 PMC4012002

