# Correction: Vol. 23, Supplement

**DOI:** 10.3201/eid2401.C32401

**Published:** 2018-01

**Keywords:** errata, erratum, correction

Axis labels for Figure 3 were incorrect in Enhancing Workforce Capacity to Improve Vaccination Data Quality, Uganda (K. Ward et al.). The corrected figure is provided here, and the article has been corrected online (https://wwwnc.cdc.gov/eid/article/23/13/17-0627_article).

**Figure 3.** Comparison of the number of doses of Penta3 recorded on different vaccine dose recording and reporting tools, Uganda. A) Doses recorded on tally sheet compared with immunization register (n = 1,664 health facilities); B) doses recorded on monthly report compared with immunization register (n = 1,686 health facilities); C) doses recorded on monthly report compared with tally sheet (n = 1,713 health facilities); D) doses recorded on the HMIS compared with monthly report (n = 1,661 health facilities; 3 outliers not shown [total no. doses >650]). p<0.001 for all comparisons. Data from sample of 2015 DQI tools; 1,667 (83%) sampled from 107 districts and 343 (17%) from a census of 7 districts. Data were missing from 2 districts. HMIS, Health Management Information System; Penta3, diphtheria/tetanus/pertussis/Haemophilus influenzae type b/hepatitis B vaccine, third dose.

